# Supplementary material for: Reduced affinity of calcium sensing-receptor heterodimers and reduced mutant homodimer trafficking combine to impair function in a model of familial hypocalciuric hypercalcemia type 1
Source: PLoS One. 2022 Jul 20;17(7):e0266993. doi: 10.1371/journal.pone.0266993 (PMC9299317; doi:10.1371/journal.pone.0266993)
Supplement: S1 Fig — (PDF) [file pone.0266993.s001.pdf]

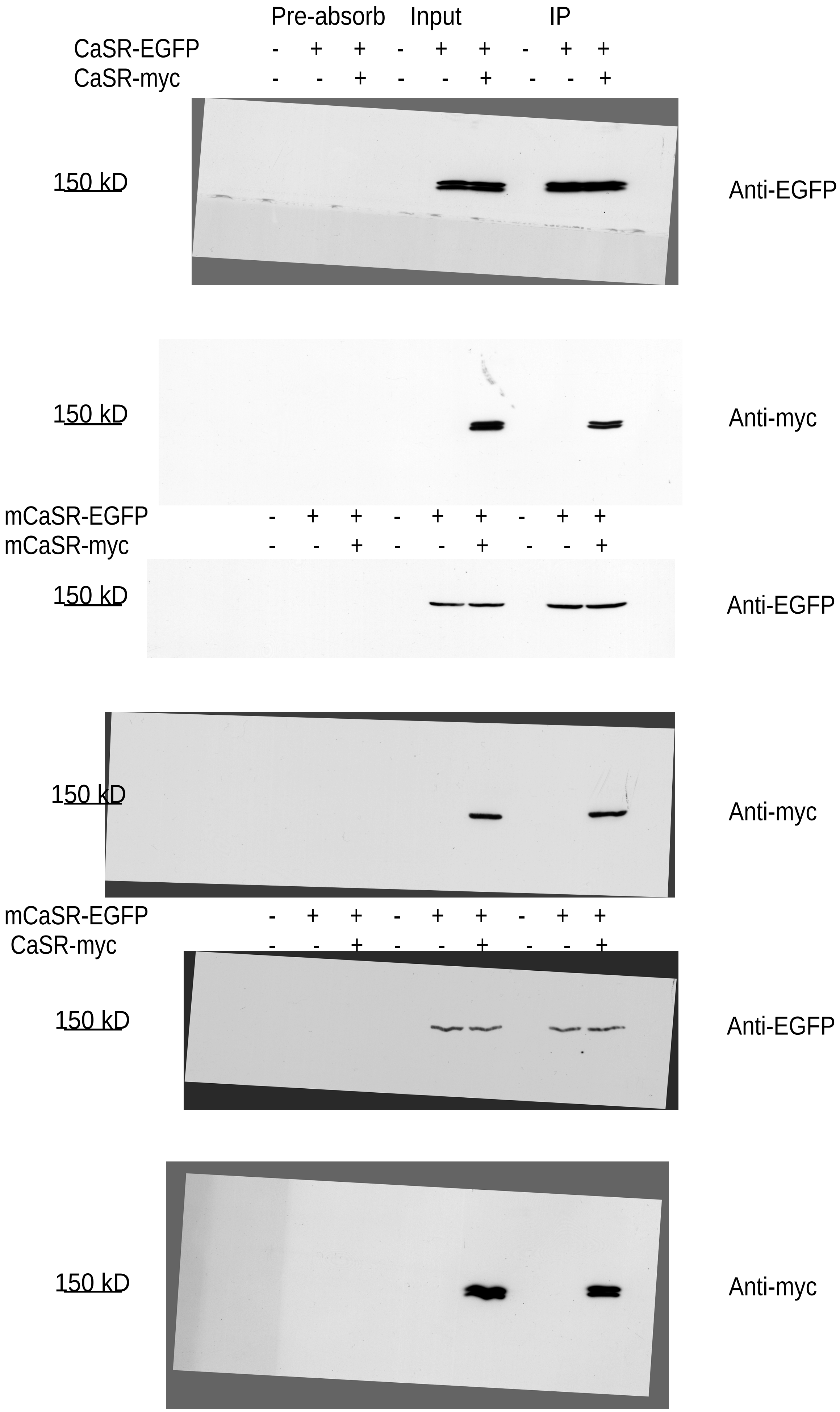

Pre-absorb
Input
IP
CaSR-EGFP - + + - + + - + +
CaSR-myc - - + - - + - - +
150 kD
Anti-EGFP
150 kD
Anti-myc
mCaSR-EGFP - + + - + + - + +
mCaSR-myc - - + - - + - - +
150 kD
Anti-EGFP
150 kD
Anti-myc
mCaSR-EGFP - + + - + + - + +
CaSR-myc - - + - - + - - +
150 kD
Anti-EGFP
150 kD
Anti-myc
